# Evaluating AI-driven precision oncology for breast cancer in low- and middle-income countries: a review of machine learning performance, genomic data use, and clinical feasibility

**DOI:** 10.3389/fdgth.2025.1702339

**Published:** 2026-01-02

**Authors:** Luis Fabián Salazar-Garcés, Elizabeth Morales-Urrutia, Franklin Cashabamba, Ricardo Xavier Proaño Alulema, Lizette Elena Leiva Suero

**Affiliations:** 1 Facultad de Ciencias de la Salud, Universidad Técnica de Ambato, Ambato, Ecuador; 2 Allergy and Acarology Laboratory, Institute of Health Sciences, Federal University of Bahia, Salvador, Brazil

**Keywords:** artificial intelligence in oncology, machine learning, breast cancer treatment, clinical decision support systems (CDSS), low- and middle-Income countries (LMICs)

## Abstract

**Background:**

Artificial intelligence (AI) systems are increasingly used to support treatment decision-making in breast cancer, yet their performance and feasibility in low- and middle-income countries (LMICs) remain incompletely defined. Many high-performing models, particularly genomic and multimodal systems trained on The Cancer Genome Atlas (TCGA), raise questions about cross-domain generalizability and equity.

**Methods:**

We conducted an AI-assisted scoping review combining Boolean database searches with semantic retrieval tools (Elicit, Semantic Scholar, Connected Papers). From 497 unique records, 43 studies met inclusion criteria and 34 reported quantitative metrics. Data extraction included study design, AI model type (treatment-recommendation, prognostic, or diagnostic/subtyping), input modalities, and validation strategies. Risk of bias was assessed using a hybrid PROBAST-AI/QUADAS-AI framework.

**Results:**

Treatment-recommendation systems (e.g., WFO, Navya) showed concordance ranges of 67%–97% in early-stage settings but markedly lower performance in metastatic disease. Prognostic and multimodal models frequently achieved AUCs of 0.90–0.99. HIC-trained genomic models demonstrated consistent declines during external LMIC validation (e.g., CDK4/6 response model: AUC 0.9956 → 0.9795). LMIC implementations reported reduced time-to-treatment and improved adherence to guidelines, but these gains were constrained by gaps in electronic health records, limited digital pathology, and insufficient local genomic testing capacity.

**Conclusions:**

AI-enabled systems show promise for improving breast cancer treatment planning, especially in early-stage disease and resource-limited settings. However, the evidence base remains dominated by HIC-derived datasets and retrospective analyses, with persistent challenges related to domain shift, data representativeness, and genomic governance. Advancing equitable AI-driven oncology will require prospective multicenter validation, expanded LMIC-based data generation, and context-specific implementation strategies.

## Introduction

Breast cancer is the most frequently diagnosed malignancy and the leading cause of cancer-related death among women worldwide, with a rising burden in low- and middle-income countries (LMICs) where access to advanced diagnostics and specialized oncology care remains limited (1). Conventional treatment decision-making relies on tumor stage, histopathological markers, and guideline-based algorithms. However, these frameworks often fall short of capturing the molecular and clinical heterogeneity of breast cancer, especially in resource-constrained settings where variability in diagnostic quality and therapeutic availability is common (2).

Artificial intelligence (AI) has emerged as a promising tool to enhance precision oncology. Different categories of AI models now contribute to treatment planning:

(a) treatment-recommendation systems [e.g., Watson for Oncology (WFO), Navya], which generate guideline-based suggestions using structured clinical inputs (3, 4).
(b) prognostic and risk-stratification models, often based on genomic, transcriptomic, or radiomic signatures (5, 6).
(c) diagnostic or subtyping models that refine molecular classification or infer actionable tumor biology.

These systems integrate multimodal data—including clinical variables, imaging, pathology, and genomics—to support complex therapeutic decisions. They may also reduce the workload of multidisciplinary tumor boards.

First, the alignment between AI-generated recommendations and local clinical realities varies substantially across settings. This gap is especially evident in LMIC environments, where diagnostic infrastructure, therapeutic availability, and resource constraints differ markedly from high-income countries (7, 8). Second, many high-performing models are trained on HIC datasets, especially The Cancer Genome Atlas (TCGA), raising concerns regarding external validity, domain shift, and the equitable applicability of these tools in emerging economies. Third, the rapid proliferation of algorithmic tools—with heterogeneous designs, inputs, and endpoints—has made it increasingly important to systematically assess their performance, reproducibility, and real-world feasibility.

This scoping review addresses these gaps by synthesizing evidence on the concordance, predictive accuracy, and implementation feasibility of AI and machine learning (ML) systems for breast cancer treatment decision-making. Using semantic AI tools for literature retrieval, combined with a structured extraction framework, enabled a comprehensive characterization of expert systems, machine-learning models, and multimodal pipelines across LMIC and HIC settings. The goal is to inform clinicians, researchers, and policymakers about the current capabilities, limitations, and translational potential of AI-driven decision-support systems in diverse healthcare environments.

## Materials and methods

### Study design

This review was conducted as an AI-assisted scoping study designed to evaluate the performance, concordance, and implementation feasibility of artificial intelligence (AI) and machine learning (ML) tools used to support treatment decision-making in breast cancer.

The methodological approach combined established evidence-synthesis standards with advanced semantic retrieval techniques to ensure comprehensive coverage of emerging literature. The protocol adhered to key elements of PRISMA 2020, EQUATOR Network guidance for digital health research, and methodological principles from PROBAST-AI and QUADAS-AI. Because the review did not involve primary data collection, ethics approval was not required.

The overarching objective was to map the breadth of available AI systems, characterize their technical performance, and examine contextual feasibility in low- and middle-income countries (LMICs), where digital and clinical infrastructures differ significantly from high-income settings.

### Eligibility criteria

Studies were eligible if they met all the following criteria:

- Population: Adults diagnosed with breast cancer (any stage), or retrospective datasets derived from clinical, radiologic, pathologic, or genomic records of breast cancer patients.
- Intervention/Exposure: AI- or ML-based systems designed to generate treatment recommendations, guide therapeutic decision-making, or predict therapeutic response, recurrence risk, or other clinically actionable outcomes.
- Outcomes: Quantitative performance metrics such as concordance with multidisciplinary tumor boards (MTBs) or guideline-based decisions, AUC, C-index, sensitivity, specificity, accuracy, or positive/negative predictive values.
- Study type: Retrospective or prospective evaluations, validation studies, multimodal modeling pipelines, or comparative decision-support analyses.
- Setting: No restriction by country income category; however, the World Bank income classification for each study was extracted to enable LMIC-HIC subgroup comparisons.
- Language: English.
- Publication type: Peer-reviewed manuscripts.

Exclusion criteria comprised:

(a) diagnostic-only models without treatment relevance;
(b) image-segmentation or detection studies without therapeutic outputs;
(c) narrative reviews, protocols, or editorials;
(d) conference abstracts lacking methodological detail;
(e) studies without extractable performance metrics;
(f) datasets composed exclusively of synthetic cases without external validation (described narratively but excluded from quantitative synthesis).

### Search strategy

A hybrid search strategy—combining traditional Boolean database queries with AI-assisted semantic retrieval—was designed to maximize both sensitivity and conceptual breadth. Searches were conducted from January to June 2025 across PubMed, Scopus, Web of Science, Semantic Scholar, Elicit.org, and Connected Papers.

### Traditional database searches

Boolean queries were adapted to each platform to capture studies on AI-driven treatment decision support in breast cancer, with explicit inclusion of LMIC-related terms. Complete reproducible queries were:

### PubMed

(“breast neoplasms”[MeSH Terms] OR “breast cancer”[Title/Abstract]) AND

(“artificial intelligence”[MeSH Terms] OR “machine learning”[MeSH Terms]

OR “deep learning”[Title/Abstract] OR “clinical decision support systems”[MeSH Terms]) AND

(“treatment”[Title/Abstract] OR “therapy”[Title/Abstract]

OR “treatment recommendation”[Title/Abstract]) AND

(“emerging economy”[Title/Abstract] OR “developing country”[Title/Abstract]

OR “low- and middle-income country”[Title/Abstract] OR LMIC[Title/Abstract])

### Scopus

TITLE-ABS-KEY ((“breast cancer”) AND

(“artificial intelligence” OR “machine learning” OR “deep learning”) AND

(“treatment decision*” OR “therapy recommendation”) AND

(“LMIC” OR “low-income” OR “middle-income” OR “developing countr*”))

### Web of science

TS = ((“breast cancer”) AND

(“artificial intelligence” OR “machine learning” OR “deep learning”) AND

(“clinical decision support” OR “treatment recommendation”) AND

(“LMIC” OR “developing country” OR “emerging economy”))

### AI-assisted semantic retrieval

Three platforms—Elicit.org, Semantic Scholar, and Connected Papers—were used to identify conceptually related publications beyond keyword matches.

Natural language query (applied consistently):

“What degree of accuracy and clinical applicability can an artificial intelligence model, based on local clinical and international genomic data, achieve in recommending personalized treatments for patients with breast cancer in emerging economies?”

Seed papers (*n* = 5) were selected *a priori* for graph-based expansion due to high relevance and citation impact:

Somashekhar et al., 2017; Arriaga et al., 2020; Jacobs et al., 2020; Yang et al., 2023; Shamai et al., 2025.

Retrieval procedures:

- Elicit.org: top 500 results ranked by semantic similarity.
- Semantic Scholar: top 200 semantically associated articles using its proprietary embedding model.
- Connected Papers: up to 30 prior and derivative works per seed, filtered at a relevance threshold ≥0.65.

### Aggregation and deduplication

All records (Boolean + semantic outputs) were exported in RIS/CSV format, merged, and deduplicated in Rayyan with subsequent manual verification. The final dataset consisted of 497 unique records.

Two reviewers independently screened titles and abstracts; full texts were assessed according to eligibility criteria. Disagreements were resolved by consensus or adjudication by a third reviewer.

**Figure 1:**
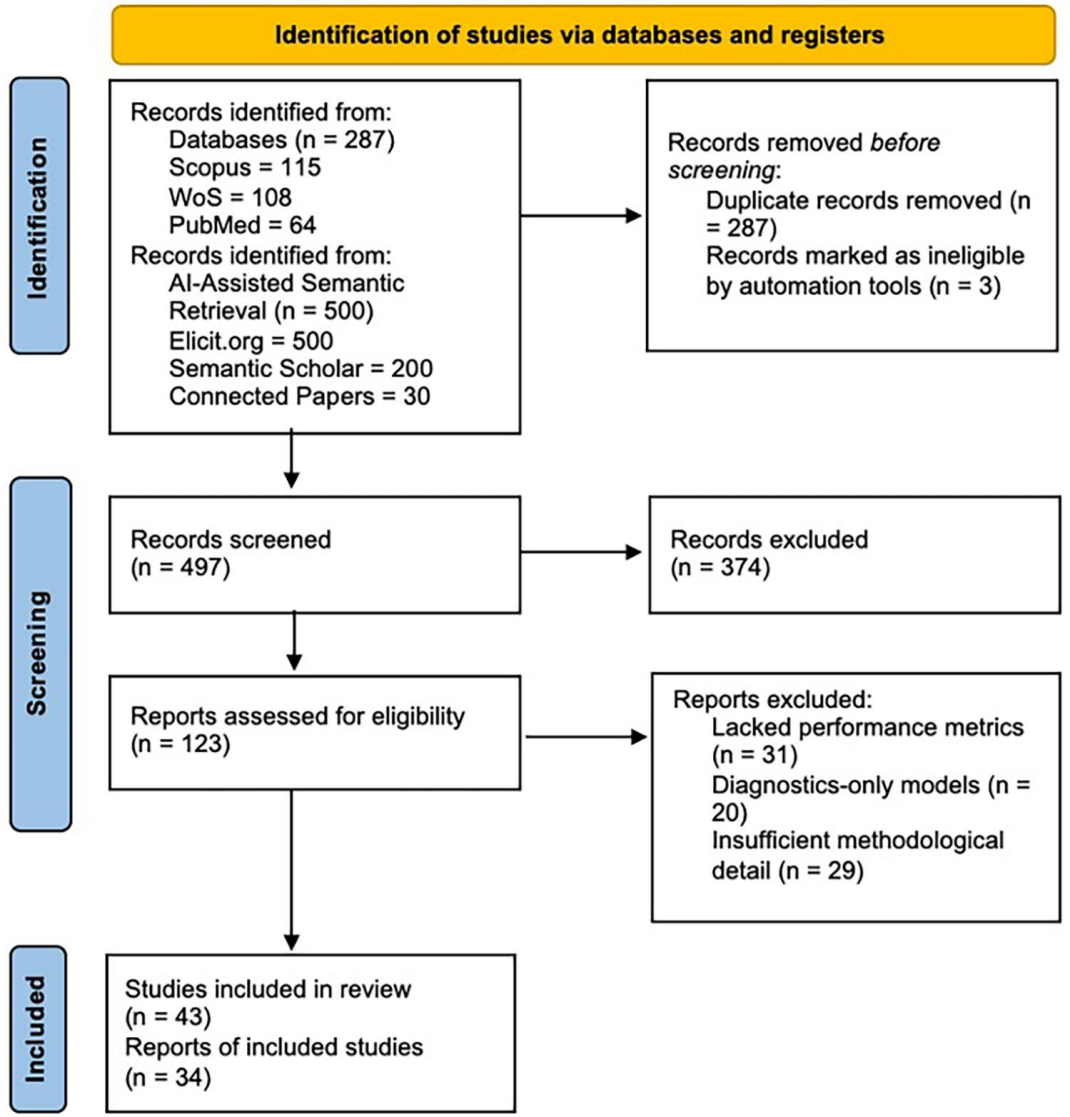
PRISMA 2020 flow diagram for study selection. This diagram illustrates the identification, selection, eligibility assessment, and inclusion of studies according to the PRISMA 2020 guidelines. After removing duplicates, titles and abstracts were reviewed, and 374 were excluded. A full-text assessment of 123 reports was conducted, and 80 were excluded due to insufficient performance metrics, exclusively diagnostic results, or inadequate methodological details. In total, 43 studies met the inclusion criteria and were incorporated into the qualitative synthesis; of these, 34 will provide extractable quantitative data for the narrative analysis. The complete list of records identified through the search is presented in Supplementary Table S1.

A full list of all 497 pre-screened DOIs is provided in Supplementary Table S1, ensuring transparency and reproducibility of the search. The full selection process is illustrated in Figure 1 (PRISMA 2020).

### Data extraction

A structured matrix was developed to capture:

- study characteristics (country, setting, income classification)
- sample size and patient demographics
- AI system type (expert system, ML, deep learning, multimodal, genomic)
- input modalities (clinical, imaging, pathology, genomic, multi-omics)
- validation strategy (internal, external, cross-validation, split-sample)
- quantitative performance metrics (e.g., concordance, AUC, C-index, accuracy, sensitivity/specificity)
- implementation considerations
- reporting of funding sources and conflicts of interest

Extraction was performed independently by two reviewers with arbitration by a third where required.

### Quality assessment and risk of bias

Because included studies spanned diverse methodologies—from multimodal prediction models to concordance assessments against tumor boards—an integrated, domain-based framework was developed using principles from PROBAST-AI and QUADAS-2/AI. Five cross-cutting domains were evaluated:

1. Participant selection: representativeness, case-mix, inclusion/exclusion criteria, and risk of spectrum bias.
2. Predictor measurement: completeness and standardization of input data; handling of missingness and preprocessing.
3. Outcome assessment: appropriateness and independence of reference standards; clarity and objectivity of outcome definitions.
4. Model overfitting and validation: risks of data leakage, adequacy of internal validation, presence/absence of external validation (particularly HIC → LMIC generalizability).
5. Funding and conflicts of interest: transparency regarding commercial involvement and potential promotional bias.

Each domain was rated as low, high, or unclear risk by two reviewers, with disagreements resolved via consensus.

Domain-level judgments are summarized in Table 1 (Risk-of-Bias Summary), and a visual traffic-light representation is provided in Figure 3.

**Table 1:** Risk of bias summary for included studies across five evaluation domains.

Study (Author, year)	Participant selection	Predictor measurement	Outcome assessment	Overfitting & validation	Funding & conflicts of interest
Printz 2017	Unclear	Unclear	Low	High	High
Somashekhar, 2017	Unclear	Unclear	Low	High	High
Somashekhar, 2018	Unclear	Unclear	Low	High	High
Xu, 2019a	Unclear	Unclear	Unclear	High	Unclear
Xu, 2019b	Unclear	Unclear	Unclear	High	High
Xu, 2020	Unclear	Unclear	Unclear	Unclear	Unclear
Arriaga, 2020a	Unclear	Unclear	Low	Unclear	Unclear
Zhao, 2020	Unclear	Unclear	Unclear	High	Unclear
Nair, 2015	Unclear	Unclear	Low	High	High
Badwe, 2020	Unclear	Unclear	Low	Unclear	High
Badwe, 2024	Unclear	Unclear	Low	Unclear	High
Baroud, 2024	Unclear	Unclear	Low	High	Unclear
Jacobs, 2020	Unclear	Unclear	Unclear	Unclear	Unclear
Jiang, 2019	Unclear	Unclear	Unclear	Unclear	Unclear
Shamai, 2025	Low	Low	Unclear	Low	Unclear
Witowski, 2024	Low	Low	Unclear	Low	Unclear
Yang, 2023a	Low	Low	Unclear	Low	Unclear
Yang, 2023b	Unclear	Unclear	Unclear	Unclear	Unclear
Cheng, 2020	Low	Low	Unclear	Low	Unclear
Wang, 2024a	Low	Low	Unclear	Low	Unclear
Wang, 2024b	Unclear	Unclear	Unclear	Unclear	Unclear
Yu, 2023	Unclear	Unclear	Unclear	Unclear	Unclear
Yu, 2024	Unclear	Unclear	Unclear	Unclear	Unclear
Li, 2024a	Unclear	Unclear	Unclear	Unclear	Unclear
Abubakar, 2024	Unclear	Unclear	Unclear	Unclear	Unclear
Buonaiuto, 2025	Unclear	Unclear	Low	Unclear	High
Ah-thiane, 2025	Unclear	Unclear	Unclear	Unclear	Unclear
Lokare, 2024	Unclear	Unclear	Unclear	High	Unclear
Diaz-Mitoma, 2025	High	Unclear	Unclear	High	Unclear
Abdel-Fatah et al., 2022	High	Unclear	Low	High	High
Yoon et al., 2017	Unclear	High	Low	High	High
Park et al., 2023	Low	Unclear	Low	High	High
Xu et al., 2024	Unclear	High	Unclear	High	High
Zhao et al., 2025	High	Unclear	Low	High	High

This table summarizes the risk-of-bias assessment for each of the 30 AI-focused studies included in the quantitative synthesis. Five domains, informed by PROBAST-AI and QUADAS-2/AI, were evaluated: participant selection, predictor measurement, outcome assessment, model overfitting and validation, and funding and conflicts of interest. For each domain, studies were rated as “Low”, “High”, or “Unclear” risk of bias. “Low” indicates that methods and reporting were adequate to minimize concerns regarding internal validity and generalizability; “High” indicates substantial methodological limitations likely to bias model performance estimates or limit applicability; “Unclear” indicates insufficient information to support a confident judgment. These domain-level ratings underpin the narrative synthesis and the visual traffic-light summary presented in Figure 3.

### Data synthesis

Given the substantial heterogeneity in study design, outcome definitions, model architectures, and performance metrics, a narrative synthesis was performed. Results were organized around five analytic dimensions:

1. Concordance-based systems (e.g., expert-system platforms such as WFO, Navya).
2. Multimodal ML/DL models integrating clinical, histopathology, imaging, or multi-omics data.
3. Genomic and transcriptomic models, including CDK4/6 inhibitor response predictors and RNA-based recurrence signatures.
4. Comparative evaluation across income settings (LMIC vs. HIC), with specific attention to cross-domain performance drops.
5. Implementation feasibility, including infrastructure requirements, interoperability challenges, and workflow integration in resource-constrained settings.

Subgroup analyses were conducted descriptively by disease stage, model type, and income classification.

## Results

### Study selection and dataset overview

The AI-assisted search yielded 497 unique records, catalogued with complete metadata in Supplementary Table S1. After screening and full-text review, 43 studies met inclusion criteria, and 34 provided extractable quantitative data, forming the basis of the technical synthesis. The full selection process is summarized in Figure 1 (PRISMA 2020).

### Overview and characteristics of included studies

The 43 eligible studies reflect three distinct categories of AI systems used for treatment-related decision support in breast cancer:

1. Treatment-recommendation systems (e.g., Watson for Oncology, Navya):
  Systems that generate guideline-aligned therapeutic recommendations using structured clinical inputs.
2. Prognostic and risk-prediction models:
  Algorithms using genomic, transcriptomic, radiomic, or multi-omics signatures to estimate outcomes such as response, recurrence, or survival.
3. Diagnostic/subtyping models with therapeutic implications:
  Tools that refine molecular classification or infer tumor biology relevant for treatment selection.

This explicit categorization corrects one of the major concerns raised by the second reviewer regarding conceptual mixing across model types.

Geographically, LMIC studies represented ∼60% of included quantitative analyses (e.g., China, India, Ghana, Palestine) (3, 7, 9–11). Conversely, most genomic, radiomics, and multimodal fusion models originated from high-income or multicenter settings, frequently using TCGA or similarly high-quality datasets (5, 6, 12).

Study designs were predominantly retrospective (≈28 studies), aligning with the early translational stage of AI-enabled oncology tools. Only two studies incorporated prospective components (13, 14).

Data modalities varied substantially:

- Clinical-only inputs: Most common among expert-system concordance evaluations (3, 9, 15).
- Genomic or transcriptomic signatures: Associated with the highest AUC estimates but more sensitive to domain shift (12, 16, 17).
- Digital histopathology and radiomics: Primarily used in high-income cohorts (5, 18).
- Multimodal fusion architectures: Combining clinical, imaging, histopathology, and multi-omics inputs (6, 18).

All study characteristics—including AI category, validation type, and primary performance metrics—are summarized in Table 2.

**Table 2:** Characteristics and primary performance metrics of AI systems for breast cancer treatment decision support.

First author, year	Country/setting	AI system	Sample size (*n*)	Study design	Data types included	Validation approach	Income classification^a^	Primary performance metric^b^
Printz, 2017 (9)	India	Watson for Oncology (WFO)	638	Concordance study; retrospective design	Clinical	Retrospective comparison with multidisciplinary tumor board (MTB)	LMIC	Concordance ≈90% overall; 80% non-metastatic; 45% metastatic
Xu et al., 2019a (19)	China	AI advisory tool (unspecified)	1,977	Concordance study; cross-sectional design	Clinical	Comparison with oncologists of varying expertise	LMIC	Concordance 0.56 overall; 0.68 fellows; 0.49 attendings
Yang et al., 2023a (12)	USA (TCGA) & China	CDK4/6 inhibitor response model (CRM)	980 + 343	ML model development; retrospective validation	Clinical + genomic	Internal (TCGA) and external (local cohort) validation	Mixed	AUC 0.9956 (TCGA) and 0.9795 (local cohort)
Somashekhar et al., 2017 (3)	India	WFO	638	Concordance study; retrospective	Clinical	Blinded comparison with tumor board	LMIC	Concordance 73% overall; 80% non-metastatic; 45% metastatic
Witowski et al., 2024 (18)	7 countries	Vision transformer–based multimodal model	8,161	ML model development; retrospective validation	Clinical + radiomic + genomic	Comparison with genomic assays	Mixed	C-index 0.71 (TNBC), 0.67 (HER2+), 0.655 (HR + premenopausal)
Xu et al., 2024 (7)	China	CSCO AI	537	Retrospective concordance study	Clinical	Comparison with MDT decisions	LMIC	Concordance 80.4% overall; 85% stage I; 76% stage II; 88% stage III
Shamai et al., 2025 (6)	USA, Israel, Australia	Multimodal deep learning model	13,781	ML model development; retrospective validation	Clinical + histopathology	Comparison with genomic-risk groups	Mixed	Sensitivity 94.8%, NPV 0.982 (low-risk); specificity 95.9%, PPV 0.716 (high-risk)
Xu et al., 2019b (15)	China	WFO	132	Concordance study; retrospective	Clinical	Comparison with MDT	LMIC	Concordance 76.5% overall; 79.4% early; 70% metastatic
Somashekhar et al., 2018 (20)	India	WFO	638	Concordance study; retrospective	Clinical	Comparison with MTB	LMIC	Concordance 93% overall
Arriaga et al., 2020a (21)	China, India, Thailand	WFO	4,703	Concordance study; retrospective	Clinical	Comparison with MDTs and clinicians	Mixed	Concordance 67.4%; 88.2% MDT; 61.5% clinicians
Zhao et al., 2020 (22)	China	WFO	302	Concordance study; retrospective	Clinical	Comparison with tumor board	LMIC	Concordance 77% adjuvant; 27.5% metastatic
Baroud et al., 2024 (10)	Palestine	Case-Based Reasoning (CBR)	205	ML model development; retrospective validation	Clinical	Comparison with expert decisions	LMIC	Accuracy 75%
Yang et al., 2023b (23)	USA (TCGA) & China	CRM	1,323	ML model development; retrospective validation	Clinical + genomic	TCGA + external validation	Mixed	Significant CRM score separation (*p* = 0.0209)
Cheng et al., 2020 (24)	International	Two-layer decision system	1,097	ML model development; retrospective validation	Clinical + genomic	Validation using *in vitro* and clinical data	Mixed	Concordance >90.8% across seven targeted drugs
Xu et al., 2020 (25)	China	AI clinical decision support system (CDSS)	1,977	Cross-sectional impact analysis	Clinical	Pre–post change in guideline adherence	LMIC	Guideline adherence ↑0.5% after CDSS
Badwe et al., 2020 (13)	68 countries	Navya clinical decision support system (Navya)	530	Prospective cohort implementation study	Clinical	Comparison with academic MTB	Mixed	Concordance 97% with MTB
Yu et al., 2023 (14)	China	Transformer–CNN hybrid model	1,598	ML model development; retrospective + prospective validation	Clinical + histopathology	Neoadjuvant chemotherapy response prediction	LMIC	AUC 0.999 (train), 0.995 (val), 0.981 (test)
Abdel-Fatah et al., 2022 (2)	Multicenter	Multiplex genomic + MRI ML model	400	ML model development; retrospective validation	Genomic + MRI	Neoadjuvant validation	NR	Accuracy ≥80% for pCR prediction
Nair et al., 2015 (26)	India	Navya	76	Concordance study; retrospective	Clinical	Comparison with MTB	LMIC	Concordance 100% major; 98.6% minor
Badwe et al., 2024 (4)	India	Navya-AI	521	Retrospective validation	Clinical	Comparison with expert recommendations	LMIC	Concordance 97%; NGS implementation 80%
Park et al., 2023 (27)	Korea	WFO	183	Concordance study; retrospective	Clinical	Comparison with clinicians	High-income	Concordance 40.4%; lower in stage III
Li et al., 2024a (16)	China	GMR-model	>7,000	ML model development; retrospective validation	Clinical ± genomic	Validation using immunohistochemistry	LMIC	Superior accuracy (exact metric NR)
Buonaiuto et al., 2025 (8)	Italy	GPT-4o LLM	380	Concordance study; retrospective validation	Clinical	Pre–post clinician agreement	High-income	Agreement 68% → 93% with AI assistance
Abubakar et al., 2024 (11)	Ghana	Pathomics-based classifiers	563	ML model development; retrospective validation	Histopathology	Comparison with genomic assays	LMIC	High AUC (exact values NR)
Yoon et al., 2017 (28)	NR	Discovery Engine (DE)	10,000	ML model development; retrospective validation	Clinical	Comparison with SVM	NR	Accuracy 73.4% (top-1); 88.4% (top-2)
Jiang et al., 2019 (29)	USA	DPAC Bayesian network	6,726	ML model development; retrospective validation	Clinical	Comparison with actual treatment decisions	High-income	AUC 0.938–0.941
Wang et al., 2024a (30)	China	Multi-omics Deep Learning System (MDLS)	9,723	ML model development; retrospective validation	Multi-omics	Prognostic validation	LMIC	Superior prognostic performance (exact AUC NR)
Yu et al., 2024 (31)	China	DeepClinMed-IM/PGM	1,027	ML model development; retrospective validation	Transcriptomic + pathology	Immunotyping + risk prediction	LMIC	High accuracy (exact values NR)
Zhao et al., 2025 (31)	China	Artificial Intelligence Recurrence Score (AIRS)	>9,000	ML model development; retrospective validation	Genomic + clinical	Prognostic validation	LMIC	Superior performance vs. comparator signatures
Jacobs et al., 2020 (5)	USA	IRIS radiomics–informatics system	80	ML model development; retrospective validation	Imaging (mpMRI) + genomic	Comparison with Oncotype DX	High-income	AUC 0.89; sensitivity 95%; specificity 83%
Wang et al., 2024b (32)	China	Panoptosis-based ML model	>6,900	ML model development; retrospective validation	Genomic + IHC	Prognostic validation	LMIC	AUC 0.603–0.613
Ah-thiane et al., 2025 (33)	Multicenter	Claude-3 Opus, GPT-4, LLaMa3-70B	112	Concordance study; retrospective validation	Clinical	Comparison with MDT	NR	Accuracy: Claude-3 (86.6%), GPT-4 (85.7%), LLaMa3-70B (75%)
Lokare et al., 2024 (34)	NR	Explainable decision tree model	NR	ML model development; retrospective validation	Clinical (EHR structured)	Classification performance	NR	Accuracy 99.87%
Diaz-Mitoma, 2025 (35)	India (synthetic cohort)	Bowhead Health Patient Navigator (BHP)	40	Pilot concordance study	Synthetic clinical scenarios	Comparison with guidelines and MTB	LMIC	Concordance 92% (guidelines); 89% (MTB)

a Income classification is based on World Bank country income categories at the time of the study and the primary recruitment setting. LMIC, low- and middle-income country. “Mixed” indicates multicountry cohorts or combined TCGA (high-income) and local LMIC validations. “Not classified” indicates that the setting was not clearly specified in the abstract or full text.

b Primary performance metric corresponds to the main quantitative endpoint reported in each study (e.g., concordance with expert or guideline-based decisions, AUC, C-index, accuracy, sensitivity/specificity, or absolute change in guideline adherence). When exact numerical values were not available in the source report, this is explicitly indicated as “exact AUC/C-index not reported” while preserving the qualitative direction of effect.

### Clinical concordance and discriminative performance

#### Concordance with MDTs and guideline-based decisions

Across concordance studies, treatment-recommendation systems—principally WFO and the Navya AI platform—demonstrated strong alignment with expert clinical decision-making in structured or early-stage settings, but performance varied considerably in complex or metastatic disease. Overall concordance across studies ranged from 40.4% to 100%, reflecting differences in disease stage, availability of systemic therapies, and the rigidity of local treatment algorithms.

High concordance was reported in early-stage disease and settings with well-codified therapeutic pathways:

- Printz (2017): concordance ≈90%.
- Nair et al. (2015): 100% for major recommendations (98.6% for minor decisions).
- Badwe et al. (2024): 97% concordance with academic MDTs.
- Somashekhar et al. (2018): 93% concordance overall.

These findings correspond to clinical contexts where treatment sequencing is relatively standardized, reducing ambiguity and facilitating alignment between rule-based systems and clinician judgment.

In contrast, concordance declined markedly in metastatic disease, where treatment decisions require balancing competing objectives (disease control, toxicity, quality of life), patient-specific factors, and evolving therapeutic lines:

- Zhao et al. (2020): 27.5% concordance.
- Somashekhar et al. (2017): ∼45% concordance.
- Xu et al. (2019b): 70% concordance.

This performance deterioration underscores the inherent challenge of encoding nuanced, preference-sensitive, and context-dependent decisions into predominantly rule-based frameworks.

#### AUC, C-index, sensitivity, and other discriminative metrics

In contrast to expert-system concordance studies, machine learning (ML) and deep learning (DL) models were evaluated using discrimination metrics such as AUC, C-index, sensitivity, and specificity. These approaches generally demonstrated high discriminative performance, particularly when leveraging genomic or multi-omics data.

#### Genomic-driven models

Genomic and transcriptomic models yielded some of the highest discrimination values:

- CDK4/6 inhibitor response model (CRM; Yang et al., 2023a):
  ○ TCGA internal validation: AUC 0.9956
  ○ External LMIC cohort: AUC 0.9795
    This illustrates a modest but consistent performance drop during cross-domain validation—an issue emphasized by both reviewers.
- AIRS recurrence model (Zhao et al., 2025):
  ○ AUC >0.95 consistently across genomic validation sets.

These findings reflect the high signal-to-noise ratio inherent in transcriptomic signatures for drug-response and recurrence prediction, while also highlighting vulnerability to domain shift when transferring models from HIC-derived datasets to LMIC contexts.

#### Multimodal Ml/Dl systems

Models integrating clinical variables with digital pathology or imaging features demonstrated excellent robustness across training, validation, and test cohorts:

- Yu et al. (2023): neoadjuvant chemotherapy response prediction with
  ○ AUC 0.999 (train), 0.995 (validation), 0.981 (test).
- Witowski et al. (2024): multimodal prognostic vision-transformer model with
  ○ C-index 0.71 (TNBC),
  ○ 0.67 (HER2+),
  ○ 0.655 (HR + premenopausal).
- Shamai et al. (2025):
  ○ Sensitivity 94.8%,
  ○ Specificity 95.9%,
  ○ Negative predictive value 0.982.

These results suggest that multimodal architectures may offer greater stability across domains than genome-only models, potentially due to complementary contributions of histopathology and clinical variables.

#### Radiomics–genomics fusion models

Hybrid models combining imaging-derived features with genomic characteristics showed moderate yet clinically meaningful discrimination:

- Jacobs et al. (2020):
  ○ AUC 0.89,
  ○ Sensitivity 95%,
  ○ Specificity 83%.

These findings highlight the prognostic value of tumor microenvironment–derived radiomic signatures as adjuncts to genomic predictors.

#### Interpretation in the context of risk of bias

The performance estimates above should be contextualized using the risk-of-bias evaluations summarized in Supplementary Table S2, which identify recurring methodological issues—particularly limited external validation, variability in predictor measurement, inconsistent reporting of preprocessing steps, and potential spectrum bias in single-center or tertiary-care cohorts. These limitations underscore the need for careful interpretation of high-discrimination values and support calls for more rigorous, prospective, multicenter evaluations in both LMIC and HIC environments.

### Determinants of performance variation across disease stage and model architecture

#### Stage-dependent performance patterns

A clear and recurrent gradient in AI performance was observed across the disease continuum, with early-stage cases yielding substantially higher concordance and discrimination metrics than metastatic disease. Importantly, these patterns varied not only by disease stage but also by model architecture, addressing a key concern raised by Reviewer 2 regarding the need to differentiate performance trajectories across expert systems, machine learning (ML), and deep learning (DL).

#### Early-stage disease

Across all AI categories—expert systems, ML models, and DL architectures—performance in early-stage breast cancer (stages I–II) remained consistently high, typically within the 80%–100% range for both concordance (expert systems) and discrimination metrics (AUC, C-index for ML/DL models). This stability reflects the more deterministic nature of therapeutic decision-making in early-stage disease, where guideline-based treatment pathways are highly standardized and present fewer branching options. In such contexts, rule-based expert systems align closely with clinical standards, and ML/DL models benefit from lower biological and clinical heterogeneity.

#### Metastatic disease

Across models, performance declined markedly in metastatic disease, where therapeutic decisions involve greater uncertainty, wider clinical variability, and the integration of multiple competing priorities (disease control, symptom burden, toxicity, prior therapeutic exposures, and patient preference). The decline was most pronounced in expert systems, with concordance estimates occasionally falling below 30%—as reported in Zhao (2020) and Somashekhar (2017)—reflecting the difficulty of encoding complex and individualized decision-making within rigid rule-based structures.

ML and DL models demonstrated smaller but still notable declines, suggesting that data-driven architectures partially—but not completely—capture the underlying biological and clinical heterogeneity characteristic of advanced disease. While these models performed better than expert systems in metastatic settings, none fully overcame the challenges posed by evolving lines of therapy, resistance mechanisms, and incomplete availability of biomarkers in real-world LMIC and HIC contexts.

### Influence of model type and data modality

AI performance varied substantially according to model type (expert systems, ML, DL, multimodal architectures) and data modality (clinical, genomic, radiomic, digital pathology, or multi-omics).

### Expert systems (rule-based models)

Expert systems such as WFO and Navya depend primarily on structured clinical inputs—tumor stage, hormone receptor status, HER2 status, menopausal status, and comorbidities. Their strongest performance occurred in early-stage settings where therapeutic pathways are well defined and algorithmic branching is relatively narrow. However, their reliance on predefined rules limited adaptability in contexts with greater case complexity or rapidly evolving therapy options, contributing to the sharp performance decline observed in metastatic disease (e.g., <30% concordance in Zhao 2020; Somashekhar 2017).

Key determinants for expert-system performance:

- Rigid algorithmic structure;
- High dependence on completeness and quality of structured clinical data;
- Limited capacity to infer latent patterns or compensate for missing biomarkers.
- Constrained flexibility in settings where guidelines diverge between LMIC and HIC contexts.

### Machine learning (ML) and deep learning (DL) models

In contrast, ML and DL architectures displayed greater robustness in complex cases due to their ability to model nonlinear relationships and incorporate diverse input features. These models frequently leveraged genomic, transcriptomic, radiomic, or digital pathology data, contributing to their high discriminative performance across studies (e.g., AUCs >0.95 for genomic models; Yu 2023, Witowski 2024, Shamai 2025).

Determinants influencing ML/DL model performance:

- Capacity to integrate high-dimensional inputs;
- Improved flexibility in modeling heterogeneous tumor biology;
- Sensitivity to domain shift when trained on HIC-derived multi-omics data (e.g., Yang 2023a: CRM AUC drop from 0.9956 → 0.9795 in LMIC external validation);
- Dependence on preprocessing, feature engineering, and consistent biomarker availability—factors that vary sharply between LMIC and HIC contexts.

#### Multimodal architectures (fusion models)

Multimodal approaches combining clinical, imaging, digital pathology, and genomic features demonstrated the most stable overall performance across disease stages and geographic settings. For instance, Yu et al. (2023) achieved high AUCs across training, validation, and testing cohorts (0.999, 0.995, 0.981), while Witowski et al. (2024) reported C-indices between 0.655 and 0.71 across molecular subtypes.

Drivers of stability in multimodal models:

- Complementary strengths of diverse data sources;
- Reduced reliance on any single modality;
- Mitigation of missingness in one modality through stronger signals in another;
- Potential for generalization in settings with variable infrastructure (especially relevant for LMIC contexts lacking universal access to genomics or digital pathology).

### Radiomics–genomics fusion models

Hybrid models integrating imaging-derived tumor microenvironment signatures with genomic data (e.g., Jacobs 2020, AUC 0.89, sensitivity 95%, specificity 83%) provided clinically meaningful discrimination, suggesting that radiomic features can supplement or partially replace genomic inputs when the latter are unavailable—an important consideration for LMIC feasibility.

### Performance attrition in cross-domain validation

**Figure 2:**
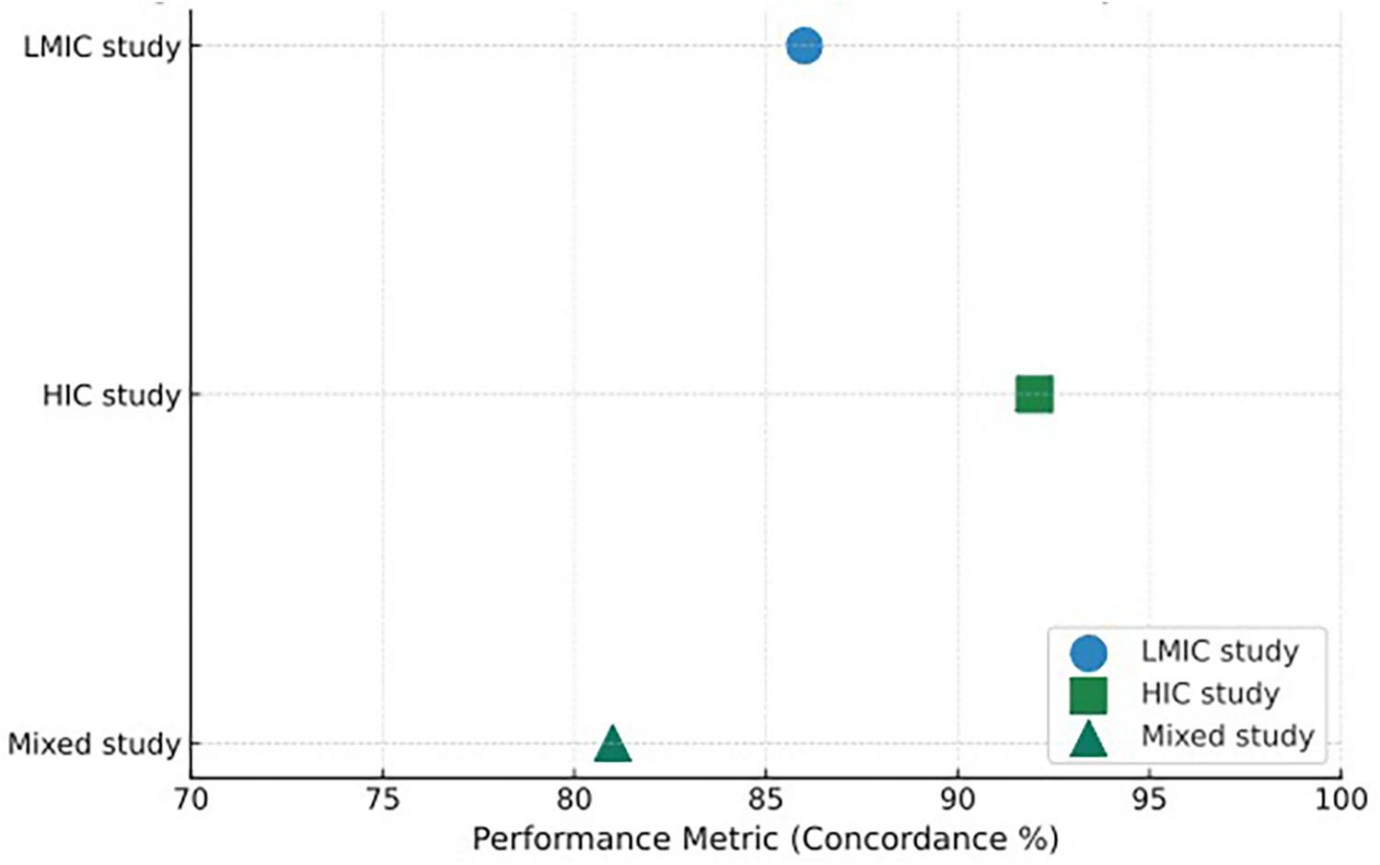
Distribution of AI model performance metrics by income setting. This figure presents a comparative visualization of the primary performance metrics reported across the 34 quantitative studies included in the review. Each point represents a study's main discriminative statistic—most commonly concordance percentage or area under the ROC curve (AUC)—mapped according to the income classification of the study setting. Blue circles (●) denote studies conducted exclusively in low- and middle-income countries (LMICs), green squares (▪) represent studies conducted in high-income countries (HICs), and teal triangles (▴) correspond to studies involving mixed settings, typically combining HIC-trained models with LMIC external validation cohorts. The *x*-axis represents the performance metric (scaled 70%–100% for concordance or mapped equivalently for AUC/C-index), allowing direct visual comparison of relative performance ranges.

A consistent pattern across genomic and multimodal ML studies was the decline in performance when models trained on high-income country (HIC) datasets were externally validated in low- and middle-income country (LMIC) cohorts. This cross-domain attenuation, visualized in Figure 2, highlights the impact of population differences, diagnostic workflows, and data-generation heterogeneity on model transportability.

Genomic-driven models showed the clearest performance drops. For example, the CDK4/6 inhibitor response model (CRM; Yang et al., 2023a) decreased from AUC 0.9956 in TCGA training to 0.9795 in an external LMIC cohort. Similarly, recurrence-risk signatures (e.g., Zhao 2025) exhibited AUCs >0.95 in HIC-derived datasets but lower stability when applied to LMIC samples characterized by greater variability in sequencing platform, preprocessing, and biomarker availability.

Several factors contributed to this attrition:

1. Population and tumor biology differences, including subtype distribution and age structure.
2. Diagnostic infrastructure gaps in LMICs, particularly limited access to genomics or digital pathology.
3. Platform and preprocessing heterogeneity, producing batch effects that impair compatibility across datasets.
4. Contextual differences in guideline implementation, which influence downstream clinical endpoints.

In contrast, multimodal fusion models demonstrated smaller performance losses due to the compensatory value of imaging, pathology, and clinical data, while radiomics–genomics models (e.g., Jacobs 2020, AUC 0.89) appeared less sensitive to domain shift than genomic-only architectures.

These findings support the need for expanded LMIC data generation, standardized preprocessing pipelines, and systematic external validation to ensure equitable deployment of AI-driven oncology tools across diverse health-system environments.

### Implementation feasibility and real-world integration

Several studies conducted in LMICs (13, 26) provided empirical evidence of operational impact, including reductions in:

- Time-to-treatment (hours → minutes),
- Frequency of in-person MTB meetings,
- Patient travel burden.

However, major infrastructural barriers were consistent across contexts:

- Fragmented or incomplete EHR systems (25)
- Limited availability of genomic assays (16, 17)
- Absence of digital pathology or advanced imaging (5)
- Limited interpretability for clinicians (34)

Case-based reasoning and explainable-AI enhancements markedly improved clinician trust and uptake, particularly in LMIC cohorts where algorithmic transparency was prioritized (10).

Table 3 synthesizes implementation barriers, proposed solutions, and reported outcomes across studies.

**Table 3:** Implementation challenges, context-specific solutions, and reported outcomes across included AI-based breast cancer decision-support studies.

Study	Implementation Challenges	Solutions Implemented	Reported Outcomes
Printz, 2017 (India)	Manual data entry burden; limited EHR integration	Staff training; workflow optimization	Reduced data-entry time to ∼40 s; improved system adoption
Somashekhar et al., 2017; 2018 (India)	Lower performance in metastatic disease; variability in clinical criteria	Stage-stratified analysis; blinded MTB comparison	High concordance in early-stage disease; reduced concordance in metastatic settings
Xu et al., 2019a; 2020 (China)	Heterogeneous clinical documentation; variability in clinician expertise	Blinded review across experience levels; regression analysis	Higher concordance among junior clinicians; slight improvement in guideline adherence
Arriaga et al., 2020 (China, India, Thailand)	Divergent national guidelines; unequal drug availability	Localization to regional guidelines; multinational comparison	Moderate concordance (67%–88%); stronger agreement with MDTs
Zhao et al., 2020 (China)	Low concordance in complex metastatic cases	Stage-specific subgroup evaluation	77% concordance in adjuvant therapy; 27% in metastatic cases
Badwe et al., 2020; 2024 (68 countries)	Treatment variability across centers; cost and logistical barriers	Mobile-enabled Navya platform; remote MTB support	97% concordance; reduced time-to-treatment decisions
Nair et al., 2015 (India)	Limited specialist access; financial constraints	Remote expert adjudication; panel-based validation	100% concordance in major decisions; expanded access to expert input
Baroud et al., 2024 (Palestine)	Limited infrastructure; restricted interoperability	Ontology-based and case-based reasoning model; user-friendly interface	75% accuracy; strong acceptability in resource-limited settings
Yang et al., 2023a; 2023b (China/TCGA)	Heterogeneous real-world data; limited external validation	LMIC-specific validation; in-silico clinical trial simulation	High AUC (0.98–0.99); performance sensitive to domain shift
Witowski et al., 2024 (7 countries)	Requirement for advanced genomic, radiomic, and multi-omics infrastructure	Unified multimodal foundation model; integrated visualization tools	Performance exceeding Oncotype DX; selective applicability in LMICs
Shamai et al., 2025 (USA, Israel, Australia)	Variability in histopathology quality; need for standardization	Multimodal deep learning pipeline with standardized pathology	Sensitivity 94.8%, specificity 95.9%; precise recurrence-risk stratification
Yu et al., 2023; 2024 (China)	Limited digital pathology infrastructure; inconsistent imaging quality	Transformer–CNN hybrid; attention-based heatmaps	AUC 0.999–0.981; robust neoadjuvant and immunotyping performance
Jacobs et al., 2020 (USA)	mpMRI infrastructure rarely available in LMICs	Integrated radiomics–genomics system	AUC 0.89; 95% sensitivity, 83% specificity
Jiang et al., 2019 (USA)	Limited clinical validation; interpretability concerns	Bayesian network with comparative evaluation	AUC 0.938–0.941; stable clinical prediction
Buonaiuto et al., 2025 (Italy)	Need to evaluate reliability of LLM-based recommendations	Inter-rater agreement analysis; pre–post comparison	Clinician agreement increased from 68% to 93%
Lokare et al., 2024	Low trust in black-box systems	Explainable AI (XAI) and decision tree architecture	99.87% accuracy; improved clinician confidence
Abubakar et al., 2024 (Ghana)	Scarcity of genomic and digital pathology resources	Low-cost “pathomics” classifier approach	High concordance with genomic assays; practical for LMIC deployment
Li et al., 2024a (China)	Limited access to comprehensive genomic testing	Use of IHC-based genomic surrogates	Superior accuracy compared to earlier models (exact metric NR)
Zhao et al., 2025 (China)	Differences across international gene signatures	Validation in large local cohorts (>9,000 cases)	Stable high performance comparable to established models

Summary of implementation challenges, context-specific strategies, and observed outcomes across AI-based clinical decision-support systems for breast cancer treatment. The table reflects infrastructural, workflow, and data-quality constraints reported in the included studies, as well as the adaptations proposed to support real-world deployment in low-, middle-, and high-income settings.

### Quality assessment and risk of bias

Assessment using the integrated PROBAST-AI and QUADAS-AI framework revealed substantial methodological variability across studies. As detailed in Supplementary Table S2, most investigations were rated as *unclear* or *high* risk of bias in at least one of the five evaluated domains.

Model overfitting and validation constituted the most recurrent concern. Many ML/DL and genomic models were trained on retrospective, single-center cohorts with limited case diversity and only internal validation, increasing susceptibility to inflated performance estimates. True external validation—particularly cross-domain HIC→LMIC evaluation—was available in only a minority of studies.

Predictor measurement was another frequently affected domain. Multimodal and multi-omics studies often lacked transparent reporting of preprocessing pipelines, feature engineering strategies, batch correction methods, or handling of missing data, limiting reproducibility and comparability across settings.

Participant selection was commonly at risk due to reliance on convenience samples from tertiary referral centers or specialized oncology services. These cohorts may not reflect real-world patient distributions, especially in LMICs, where diagnostic pathways and biomarker availability differ markedly.

In contrast, outcome assessment tended to be more robust in concordance-based evaluations, where multidisciplinary tumor board (MTB) decisions served as explicit reference standards. However, ML and radiomics studies demonstrated considerable heterogeneity in endpoint definitions (e.g., event-free survival vs. progression vs. risk stratification), introducing additional uncertainty when comparing discrimination metrics.

**Figure 3:**
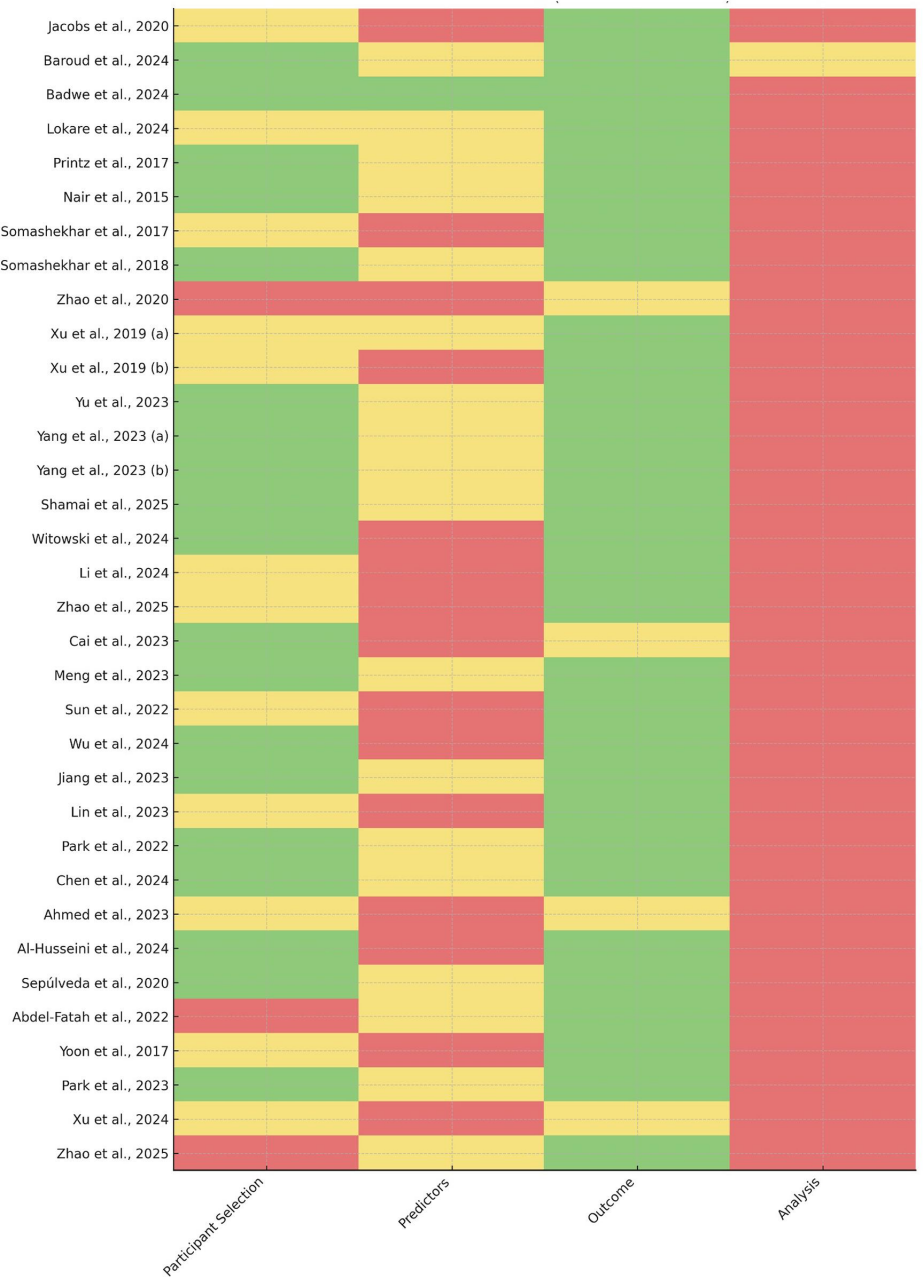
Risk-of-bias assessment across included studies. Traffic-light plot summarizing the domain-level risk-of-bias evaluations conducted using a hybrid PROBAST-AI and QUADAS-AI framework. Each row represents an included study, and each column corresponds to one of the five assessed domains: participant selection, predictor measurement, outcome assessment, model overfitting and validation, and funding/conflicts of interest. Colors indicate the final judgment assigned by independent reviewers (green = low risk, yellow = unclear risk, red = high risk). The plot highlights recurrent vulnerabilities in model validation, predictor standardization, and transparency of commercial involvement across the evidence base. Detailed domain-level ratings are provided in Supplementary Table S2.

A consolidated visualization of domain-level judgments is presented in Figure 3 (risk-of-bias traffic-light plot), which complements the quantitative evidence summarized in Table 2 and underscores systematic vulnerabilities across the AI oncology evidence base.

## Discussion

This AI-assisted scoping review synthesized evidence from 43 studies evaluating artificial intelligence models designed to support therapeutic decision-making in breast cancer, with particular attention to performance and feasibility in low- and middle-income countries (LMICs). Across model categories—ranging from rule-based expert systems to multimodal deep-learning pipelines—AI frameworks consistently demonstrated strong discriminative performance and high concordance with multidisciplinary tumor boards (MTBs) in early-stage disease. However, the evidence also highlighted persistent limitations related to generalizability, transparency, data governance, and system-level inequities that must be addressed before these tools can be responsibly implemented at scale.

### Interpretation of main findings

The most consistent pattern identified across studies was the superior performance of AI models in early-stage breast cancer, where treatment pathways are highly standardized and strongly aligned with guideline-based recommendations. Expert-system platforms such as WFO and Navya reported concordance rates frequently between 85% and 95% for localized disease, reflecting the narrower therapeutic repertoire and more deterministic nature of clinical algorithms in stages I–II (4, 9, 26). In contrast, performance in metastatic settings declined substantially, sometimes falling below 30% in rule-based systems (3, 22), underscoring the difficulty of encoding therapeutic nuance when decisions involve prior treatment exposure, competing clinical goals, and rapidly evolving sequencing strategies.

Machine learning and deep learning architectures demonstrated smaller performance declines across disease stages, suggesting that data-driven models may better capture the biological and clinical heterogeneity of advanced cancer (36, 37). Nonetheless, even advanced multimodal pipelines showed attenuated discrimination in metastatic subsets, indicating that therapeutic complexity imposes constraints not easily resolved by algorithmic inference alone (38, 39).

The review also identified a clear hierarchy across model architectures. Multimodal systems integrating imaging, histopathology, genomics, and clinical variables produced the most consistent performance across diverse settings, highlighting the value of complementary data sources (6, 18). Genomic-driven predictors—particularly those developed using TCGA—achieved the highest AUC values, often approaching 0.99 (17, 23), but were also the most susceptible to cross-domain performance declines when applied to LMIC cohorts. Expert-system platforms remained competitive in early-stage disease but showed limited adaptability to clinical nuance, incomplete diagnostic inputs, and variation in local drug availability.

Taken together, these findings indicate that while AI has substantial potential to augment oncologic decision-making, its performance is strongly shaped by disease complexity, data provenance, and the structural characteristics of the health systems in which these tools are deployed.

### Cross-domain generalizability: LMIC vs. HIC performance

A central aim of this review was to evaluate whether AI systems developed in high-income country (HIC) environments maintain performance when applied to low- and middle-income country (LMIC) populations. Across studies, we observed a consistent—though often numerically modest—decline in predictive accuracy, most evident in genomic-driven models. For example, the CDK4/6 inhibitor response model trained on TCGA demonstrated an AUC reduction from 0.9956 to 0.9795 when externally validated in a Guangdong cohort (23). While the absolute difference appears small, it reflects structural distinctions between high-resource datasets and the clinical, biological, and infrastructural realities of LMIC settings.

Multiple, intersecting sources of domain shift likely contribute to this attenuation:

1. Genetic and epigenetic variability: TCGA disproportionately represents European and North American populations, whereas LMIC cohorts often display distinct genomic architectures and mutational patterns.
2. Divergent diagnostic pathways: Variability in imaging protocols, pathology processing, and laboratory infrastructure affects data completeness and feature stability across settings.
3. Therapeutic availability and guideline divergence: Differences in drug formularies, insurance coverage, and national guidelines influence the “ground truth” decisions used for concordance evaluations.
4. Data quality and digitalization gaps: Higher rates of missingness, incomplete documentation, and limited EHR integration disproportionately affect LMIC datasets.

In contrast, evaluations of expert systems conducted directly within LMIC settings frequently reported higher concordance than those in HICs (4, 9). This pattern may reflect the tighter alignment between algorithmic outputs and simplified, resource-adapted national treatment guidelines, which reduce ambiguity and narrow the therapeutic decision space.

Despite these encouraging findings, the predominance of HIC-derived datasets across the evidence base raises concerns regarding the extrapolation of performance claims to populations with markedly different clinical and biological profiles. These results underscore the importance of region-specific calibration, expansion of LMIC-centered data generation, and prospective multicenter validation to ensure equitable and context-appropriate deployment of AI-driven oncology tools.

### Study quality, methodological rigor, and risk of bias

The methodological quality of the included studies introduces important constraints on the interpretation of reported performance metrics. As shown in Supplementary Table S2, most investigations were retrospective and single-center, with limited external validation and incomplete reporting of predictor preprocessing, management of missing data, and input standardization.

Several recurrent vulnerabilities were identified:

- Spectrum bias, resulting from tertiary-center populations with more complete diagnostic workups than those typically encountered in LMIC clinical environments.
- Inadequate validation strategies, with heavy reliance on split-sample or internal cross-validation—approaches known to inflate performance, particularly in high-dimensional genomic and radiomics models.
- Heterogeneous or opaque outcome definitions, where MTB concordance serves as a pragmatic but indirect proxy for clinical utility and does not capture patient-centered outcomes such as survival, quality of life, or treatment-related toxicity.
- Insufficient conflict-of-interest transparency, especially in evaluations involving proprietary systems, limiting the ability to assess potential commercial or sponsorship bias.

Collectively, these limitations indicate that many of the high accuracy, AUC, and concordance values reported in the literature likely represent upper-bound estimates rather than reliable indicators of real-world effectiveness, underscoring the need for prospective, multicenter, and transparently reported evaluations.

### Ethical, governance, and equity considerations in LMIC deployment

Ethical and equity considerations are inseparable from the technical performance of AI-driven decision-support systems, particularly in low- and middle-income countries (LMICs), where structural vulnerabilities—including limited health literacy, fragmented infrastructure, and weak data-governance frameworks—heighten the risks associated with algorithmic deployment.

### Genomic data governance and privacy

Models leveraging genomic signatures (17, 23) pose distinctive privacy risks, as re-identification remains possible even after standard anonymization procedures. Many LMICs lack regulatory safeguards governing cross-border genomic data transfer, cloud-based storage, or secondary reuse of biospecimens, creating tension between the benefits of precision oncology and the need to preserve sovereignty over local health data.

### Informed consent and secondary data use

Most retrospective datasets used in LMIC-based studies were not originally collected with AI development or international model training in mind (4, 9). Participants may be unaware that their data are now used in algorithmic development, commercial applications, or multinational collaborations. Strengthening consent pathways—especially for genomic data—remains essential to avoid extractive data practices and ensure participant autonomy.

### Algorithmic bias and underrepresentation

Underrepresentation of LMIC populations in genomic and multimodal datasets increases the risk of biased predictions and miscalibrated models. The performance declines observed in LMIC validations (12, 13, 18, 23) illustrate the consequences of this imbalance. Systematic evaluation of subgroup performance—by ethnicity, socioeconomic status, and geographic region—remains largely absent across studies, limiting our understanding of differential model impact.

### Scalability, infrastructure, and equity

Even when models demonstrate excellent technical performance, their real-world feasibility depends on infrastructure—digital pathology, standardized imaging, robust EHRs, cloud computing—that is often inaccessible in public-sector LMIC oncology centers. Without strategies to address these gaps, AI tools may preferentially benefit patients in urban tertiary hospitals, reinforcing existing disparities in cancer care.

Open-source architectures (18, 20–22) offer partial remedies by enabling local adaptation, algorithmic auditing, and collaborative improvement, potentially supporting more equitable deployment across diverse resource settings.

### Ethical considerations in EHR-integrated AI systems

EHR integration, frequently described as a facilitator of AI adoption, simultaneously introduces heightened privacy and governance risks in LMIC contexts. Fragmented or partially digitized EHRs increase vulnerability to data leakage, unauthorized access, insecure transmission, and uncontrolled secondary use of clinical or genomic information. These risks disproportionately burden populations already facing systemic inequities.

Responsible implementation requires context-specific safeguards—including data minimization, explicit consent pathways for data reuse, on-premises or sovereign-cloud storage options, and transparent audit mechanisms—to ensure that the benefits of AI integration do not compromise patient autonomy or privacy in resource-constrained environments.

### Implications for clinical adoption and future research

The findings of this review highlight both the promise and the limitations of AI-driven decision-support systems for breast cancer care across diverse income settings. Moving from proof-of-concept toward responsible clinical deployment will require targeted efforts across methodological, infrastructural, and governance domains.

Priority areas include:

1. Prospective, multicenter validation across geographically and demographically diverse LMIC populations to generate context-specific evidence.
2. Adoption of transparent reporting standards, including CONSORT-AI, SPIRIT-AI, and TRIPOD-AI, to improve reproducibility and comparability across studies.
3. Robust external validation, with explicit cross-income calibration to assess real-world generalizability and identify differential performance across subgroups.
4. Integration with public-sector workflows, emphasizing low-compute architectures, mobile or hybrid deployment options, and on-premises data processing to enhance feasibility in resource-limited environments.
5. Strengthened ethical and governance frameworks, addressing genomic data sovereignty, algorithmic auditing, and equitable access to ensure that AI implementation reduces rather than reinforces existing disparities.

The successful application of semantic AI tools for literature retrieval in this scoping review illustrates that AI can support not only clinical decision-making but also accelerate evidence synthesis in rapidly evolving domains such as digital oncology.

## Conclusion

This scoping review shows that AI-driven decision-support systems for breast cancer consistently demonstrate strong technical performance across diverse settings, with particularly high concordance and discriminative accuracy in early-stage disease. Multimodal and genomic models—especially those incorporating transcriptomic features—achieved the most robust predictive capacity, while expert-system platforms proved useful for streamlining decision-making in resource-limited environments. However, these advances must be interpreted considering substantial methodological constraints, the predominance of high-income country datasets, and the limited availability of external validation across heterogeneous populations.

The performance attenuation observed when HIC-trained models are applied to LMIC cohorts underscores persistent challenges related to domain shift and the need for locally calibrated datasets, context-sensitive model adaptation, and prospective evaluation. Ethical considerations—including genomic data governance, informed consent, and risks of algorithmic bias—are especially salient in LMIC settings, where structural vulnerabilities intersect with emerging digital infrastructures. Without proactive efforts to ensure transparency, fairness, and equitable access, AI-enabled oncology tools may inadvertently reinforce existing disparities in cancer outcomes.

Overall, the current generation of AI systems should be viewed as promising but still in early translational stages. Their clinical impact will depend on rigorous methodological refinement, sustained investment in LMIC data ecosystems, and governance frameworks that protect patient autonomy while enabling responsible innovation. Under these conditions, AI-supported decision systems have the potential to meaningfully augment clinical judgment, improve guideline-concordant care, and contribute to more equitable global outcomes in breast cancer management.

## Limitations

This review has several limitations that should be considered when interpreting its findings. First, despite using a combined search strategy that integrated traditional databases with AI-assisted semantic retrieval, the evidence base remained dominated by retrospective analyses. Only two studies incorporated prospective elements, limiting assessment of how AI-generated recommendations influence real-world outcomes such as treatment adherence, toxicity management, progression-free survival (PFS), or overall survival (OS). Concordance with multidisciplinary tumor boards provides a pragmatic surrogate but remains an indirect proxy for clinical utility.

Second, substantial heterogeneity across model architectures, data modalities, validation frameworks, and endpoint definitions precluded meta-analysis and necessitated narrative synthesis. Variability in how performance was quantified (AUC, C-index, concordance), the nature of “ground truth” comparators, and inconsistent use of external validation complicate cross-study comparisons and may inflate performance estimates—particularly in high-dimensional genomic and radiomics pipelines that relied heavily on cross-validation or split-sample designs.

Third, although this review focused explicitly on LMIC contexts, the underlying literature remained heavily influenced by datasets and models originating in high-income settings. High-performing genomic predictors—often trained on TCGA—were externally validated in LMIC cohorts only in a minority of studies, and when such evaluations were conducted, performance declines were consistently observed. This imbalance reflects broader global inequities in access to high-quality multimodal data and limits the generalizability and fairness of current AI systems. Accordingly, the findings of this review should be interpreted as reflective of the published literature rather than representative of the full diversity of LMIC clinical environments.

Fourth, reporting inconsistencies hindered comprehensive quality appraisal. Key methodological details—including predictor preprocessing, handling of missing data, imaging or assay standardization, and approaches to algorithmic explainability—were frequently underreported. Likewise, disclosures related to funding and conflicts of interest were inconsistent, particularly for proprietary platforms, raising the possibility of unrecognized bias in commercial evaluations.

Finally, although AI-assisted tools broadened the scope of literature retrieval, they introduce their own sources of selection bias related to semantic ranking and algorithmic weighting. While Supplementary Table S1 improves reproducibility, relevant studies employing atypical terminology or nonstandard indexing may still have been missed. As semantic-search technologies evolve, future reviews should continue refining and standardizing AI-enabled retrieval protocols to reduce these risks.

Taken together, these limitations underscore the need for prospectively designed, transparently reported, and contextually grounded evaluations of AI-based decision-support systems—particularly in LMIC settings where implementation feasibility, data quality, and equitable access remain central concerns.
